# Rapid Emergence of Highly Pathogenic Avian Influenza Subtypes from a Subtype H5N1 Hemagglutinin Variant

**DOI:** 10.3201/eid2105.141927

**Published:** 2015-05

**Authors:** Erik de Vries, Hongbo Guo, Meiling Dai, Peter J.M. Rottier, Frank J.M. van Kuppeveld, Cornelis A.M. de Haan

**Affiliations:** Faculty of Veterinary Medicine, Utrecht University, Utrecht, the Netherlands.

**Keywords:** H5N1, subtype, hemagglutinin, variant, influenza, viruses, highly pathogenic, HPAI, avian influenza, rapid emergence, evolution, monophyletic group, clade 2.3.4, reassortment events, virulent, reassortant

## Abstract

In 2014, novel highly pathogenic avian influenza A H5N2, H5N5, H5N6, and H5N8 viruses caused outbreaks in Asia, Europe, and North America. The H5 genes of these viruses form a monophyletic group that evolved from a clade 2.3.4 H5N1 variant. This rapid emergence of new H5Nx combinations is unprecedented in the H5N1 evolutionary history.

A highly pathogenic avian influenza (HPAI) A(H5N1) virus (A/goose/Guangdong/1/1996) was first detected in China in 1996. Multiple clades, defined by phylogenetic characterization of the H5 hemagglutinin (HA) (*1*), have evolved and spread across Asia, Africa, and Europe, causing enormous losses to the poultry industry. A total of 694 human infections (death rate 58%) were recorded during 2003–2014 (*2*). 

During the evolution of HPAI H5N1 viruses, reassortment events involving the 6 internal gene segments have often been detected (reviewed in [*3*]), but novel subtypes (i.e., combinations of HPAI H5 with other N subtypes) have rarely been isolated. In 2014, a novel highly virulent reassortant HPAI H5N6 virus (*4*) caused multiple outbreaks in Southeast Asia and 1 lethal human infection, which led the Food and Agricultural Organization of the United Nations to issue a warning (*5*). Outbreaks of novel HPAI H5N8 virus in South Korea (*6**,**7*), China (*8*), and Japan raised further concern, and in November 2014, this subtype emerged outside Eastern Asia, causing outbreaks in poultry farms in Germany, the Netherlands, the United Kingdom, Canada, and the United States.

## The Study

**Figure 1:**
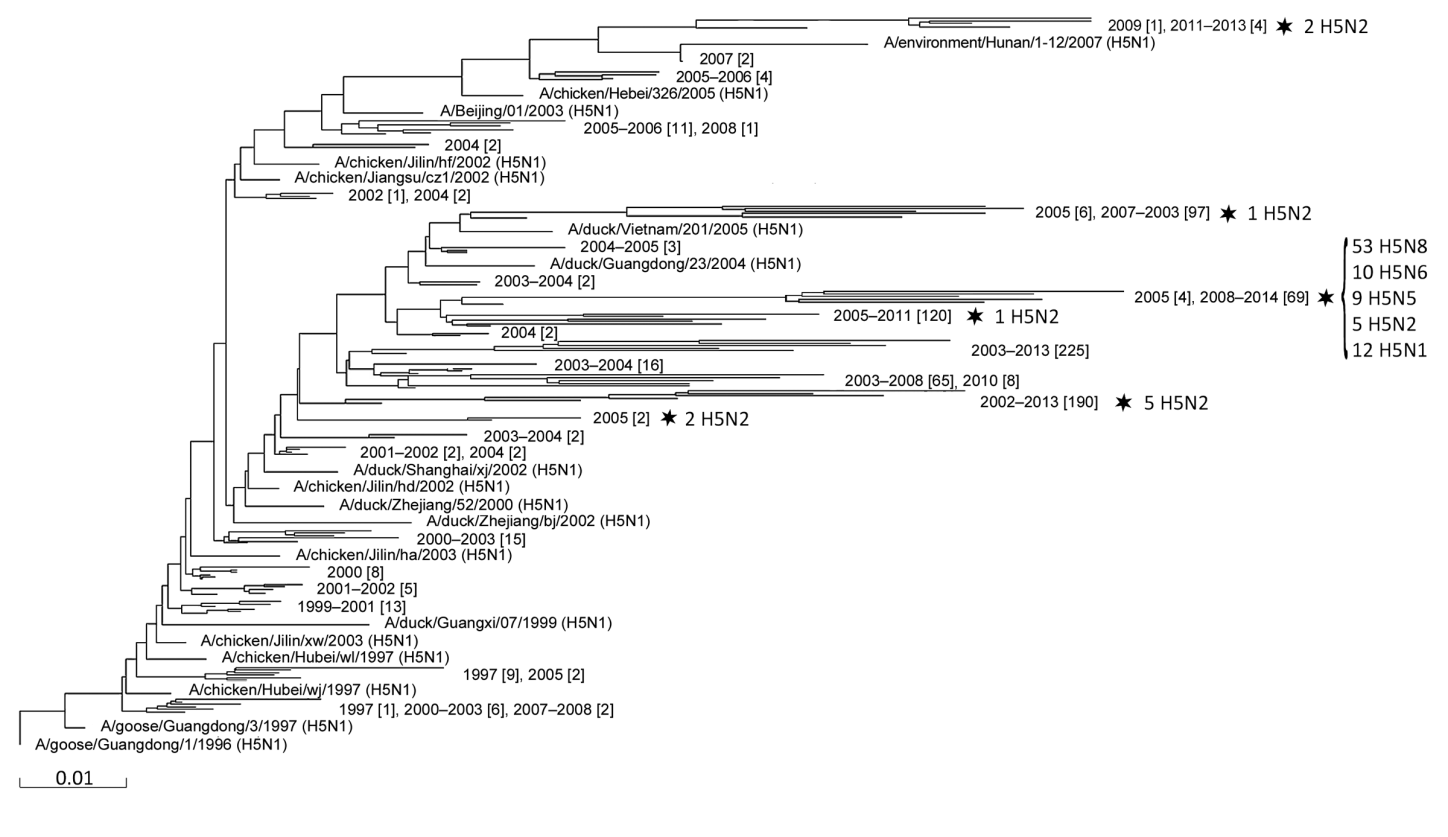
Phylogenetic tree showing the evolutionary history of the hemagglutinin (HA) proteins of novel highly pathogenic avian influenza (HPAI) H5 HA subtype viruses. By using MUSCLE (*9*), we aligned the coding region sequences for 89 HPAI H5 HA subtype viruses, excluding H5N1, with those for 850 H5N1 HA viruses representing all HPAI H5N1 clades (*1*); the 89 H5 HA sequences were identified in the NCBI Influenza Virus Resource (*10*) and the GISAID EpiFlu Database (http://www.gisaid.org). A phylogenetic tree was constructed by using the PHYLIP Neighbor Joining algorithm using the F84 distance matrix (http://www.ncbi.nlm.nih.gov/genomes/FLU/DatasetExplorer/fluPage.cgi?pageInclude=References.inc#PHYLIP). The number of sequences present in a branch is indicated between brackets. Stars indicate the branches that contain subtypes other than H5N1. The genotypes (H5N2, H5N5, H5N6, and H5N8) and their numbers of occurrence in a particular branch are indicated at right. Scale bar indicates evolutionary distance (nucleotide substitutions per site). Details for GISAID-derived sequences are shown in the Table.

To determine the evolutionary history of the HA proteins of these novel HPAI subtypes, we collected all HPAI H5 coding region sequences for all subtypes, except H5N1, and then aligned them with 850 H5N1 HA sequences representing all HPAI H5N1 clades (selected from ≈5,000 total sequences) and constructed a phylogenetic tree (Figure 1). Reassortment events leading to the generation of novel H5Nx subtypes are almost uniquely restricted to a single branch of the tree; the branch contains all isolates of the recent HPAI H5N2, H5N5, H5N6, and H5N8 outbreaks. The only other H5Nx reassortants that have been identified are a limited number of H5N2 subtype isolates that are present in 5 other branches of the tree.

**Table:** Details for GISAID-derived sequences of the hemagglutinin genome segment of various influenza A(H5) subtype viruses descended from a highly pathogenic avian A(H5N1) virus hemagglutinin variant*

Segment ID	Country	Collection date	Isolate name	Originating/submitting laboratory
EPI544756	Germany	2014 Nov 04	A/turkey/Germany-MV/R2472/2014	Friedrich-Loeffler-Institut
EPI530063	China	2013 Dec 02	A/environment/Shenzhen/25–24/2013	BGI-Shenzhen
EPI533583	China	2014 Apr 21	A/Sichuan/26221/2014	WHO Chinese National Influenza Center
EPI548623	Netherlands	2014 Nov 15	A/chicken/Netherlands/14015531/2014	Central Veterinary Institute
EPI547678	Netherlands	2014 Nov 14	A/Chicken/Netherlands/14015526/2014	Central Veterinary Institute
EPI530054	China	2014 Jan 10	A/duck/Jiangxi/95/2014	BGI-Shenzhen
EPI548493	Japan	2014 Nov 18	A/duck/Chiba/26–372–61/2014	National Institute of Animal Health
EPI548485	Japan	2014 Nov 18	A/duck/Chiba/26–372–48/2014	National Institute of Animal Health
EPI547673	UK	2014 Nov 14	A/duck/England/36254/14	Animal and Plant Health Agency
EPI543002	China	2014 Jan 20	A/duck/Beijing/FS01/2014	Institute of Microbiology, Chinese Academy of Sciences
EPI542617	China	2013 Nov 10	A/duck/Beijing/FS01/2013	Institute of Microbiology, Chinese Academy of Sciences
EPI431456	China	2011 Dec 07	A/duck/Hebei/3/2011	Institute of Microbiology, Chinese Academy of Sciences
EPI431448	China	2011 Dec 01	A/duck/Hebei/2/2011	Institute of Microbiology, Chinese Academy of Sciences

*The GISAID EpiFlu Database is available at http://www.gisaid.org. BGI, Beijing Genomics Institute; ID, identification; UK, United Kingdom; WHO, World Health Organization.

**Figure 2:**
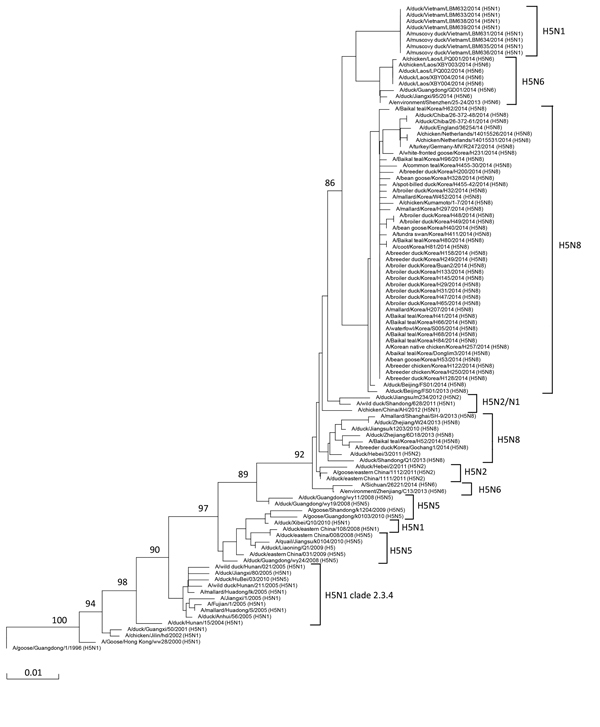
Hemagglutinin protein tree (neighbor-joining, point accepted mutation distance matrix model) of subtypes present in branch descending from highly pathogenic avian influenza A(H5N1) cluster 2.3.4 (see Figure 1). MUSCLE (*9*) was used to align protein sequences. Subtype group positions are indicated at right. Bootstrap values (n = 1,000) at key nodes are indicated. Scale bar indicates evolutionary distance (nucleotide substitutions per site).

A more detailed analysis (Figure 2) revealed that the monophyletic H5 clade harboring all the recent novel H5Nx reassortants evolved from early members of H5N1 clade 2.3.4 (a group of highly similar H5N1 viruses isolated in China in 2005). On January 12, 2015, the World Health Organization recommended designation of the novel H5 clade as 2.3.4.4 in anticipation of a revised H5 nomenclature (*11*). A previously described (*12*) H5N5 virus (A/duck/Guangdong/wy24/2008) is the first detected reassortant subtype within this clade; the donor of the NA segment of this virus could not clearly be identified (*13*). Subsequent reassortment events between viruses harboring an HA segment originally derived from the novel H5N5 viruses and a range of other avian influenza viruses have generated the H5N2, H5N6, and H5N8 subtypes. 

The HA protein of A/wild duck/Hunan/211/2005, a member of H5N1 clade 2.3.4, is highly similar to that of other clade 2.3.4 members, differing from the HA of the earliest known H5N5 descendant (A/duck/Guangdong/wy24/2008, clade 2.3.4.4) at only 12 aa positions. Seven amino acid substitutions are subsequently maintained in all descending viruses: K86R, T160A, N187D, K222Q, S227R, N244H, and A267T. Substitutions K222Q and S227R are unique to clade 2.3.4.4 and have not been observed previously in any HPAI H5N1 viruses.

Within the subtree shown in Figure 2, the H5N2 viruses are present in 2 branches. N2 of A/duck/Jiangsu/m234/2012 was derived from an H11N2 virus (*14*); the N2 of the other viruses in this branch were derived from an avian H3N2 virus (*15*). In addition, 12 H5N1 reassortants were found to be spread over different branches of the subtree (Figure 2). The N1 proteins of these reassortants are derived from different H5N1 viruses that descended from H5 clade 2.3.2. Whereas the N1 of 8 identical isolates from Vietnam (A/Muscovy duck/Vietnam/LBM631/2014) is highly similar to the N1 of clade 2.3.2.1b virus A/barn swallow/Hong Kong/1161/2010 (*1*), the N1 of A/duck/eastern China/108/2008 is highly similar to the N1 of clade 2.3.2.1c virus A/duck/Hunan/8/2008 (*1*), suggesting that independent reassortment events have taken place.

The H5N8 and the H5N6 viruses have segregated into 2 branches. Analysis of the N6 proteins (data not shown) indicates that the H5N6 viruses (all from southern China) are the result 2 independent reassortment events with avian H6N6 strains. Unfortunately, sequences from the recent H5N6 outbreaks in Vietnam (*5*) are not yet present in the databases. The 2 different H5N8 virus clusters most likely evolved from a single H5N8 reassortant virus (A/duck/Jiangsu/k1203/2010) that was isolated in China in 2010 (*13*). Both clusters were identified in Korea in 2014, whereas members of the most evolved cluster were detected later in 2014 in Japan, Germany, the Netherlands, and the United Kingdom.

## Conclusion

Since 1996, reassortment events involving H5N1 HPAI viruses have, as far as detected, only rarely led to the generation of new H5Nx subtypes. The 2008 generation of an H5N5 reassortant virus (prototype A/duck/Guangdong/wy/24/2008) represents the creation of a new HPAI virus that has led to the generation of a range of novel H5Nx reassortants that acquired novel NA proteins (H5N2, H5N6, and H5N8). The H5N6 reassortant became of particular concern after spreading over a wide geographic area in Southeast Asia and causing a fatal human infection in China (*5*). Meanwhile, the H5N8 subtype spread to Europe in November 2014, resulting in large economic losses in the poultry industry. On the basis of reports from the World Organisation for Animal Health, H5N8 and H5N2 viruses were detected in Canada and the United States in December 2014.

In this study, we exclusively focused on the unique occurrence of new HA–NA combinations. Recent publications have already described the reassortment events of the internal gene segments of several of the viruses mentioned above (*6*–*8**,**11*–*14*). In contrast to novel HA–NA combinations, novel constellations of internal gene segments are far from unique and have frequently been observed for HPAI H5N1 viruses (*3*). Our analysis indicates that new HPAI viruses have emerged that carry H5 proteins capable of matching with multiple NA subtypes. Whether the formation of new HA–NA combinations confers a selective advantage that contributed to the emergence of these novel subtypes is not known and requires elaborate research. However, the balance between HA (receptor binding) and NA (receptor cleavage) protein activities is known to be critical to cell entry and host tropism and may be an important factor that lead to the emergence of new HA–NA combinations. In contrast to HPAI H5N1, the novel clade 2.3.4.4 viruses, excluding H5N6 viruses, have not caused human infections. However, it is unknown to what extent the repeated acquisition of a new NA proteins could enhance the rate of evolution of the HA protein. Obviously such changes could further affect host and tissue specificity, potentially having serious consequences. Therefore, surveillance is required to monitor further spread, evolution, and potential changes in host range.
